# Understanding the incidence of atrial fibrillation and stroke in hypertrophic cardiomyopathy patients: insights from Danish nationwide registries

**DOI:** 10.1093/europace/euae177

**Published:** 2024-06-25

**Authors:** Christopher R Zörner, Anne-Marie Schjerning, Morten Kvistholm Jensen, Alex Hørby Christensen, Jacob Tfelt-Hansen, Jacob Tønnesen, Lise Da Riis-Vestergaard, Charlotte Middelfart, Peter Vibe Rasmussen, Gunnar Gislason, Morten Lock Hansen

**Affiliations:** Department of Cardiology, Herlev-Gentofte University Hospital, Copenhagen, Denmark; Department of Cardiology, Herlev-Gentofte University Hospital, Copenhagen, Denmark; Department of Cardiology, Rigshospitalet, Copenhagen, Denmark; The Danish Heart Foundation, Copenhagen, Denmark; Department of Cardiology, Aarhus University Hospital, Aarhus, Denmark; Department of Cardiology, Herlev-Gentofte University Hospital, Copenhagen, Denmark; Department of Cardiology, Rigshospitalet, Copenhagen, Denmark; Department of Clinical Medicine, Faculty of Health and Medical Sciences, University of Copenhagen, Copenhagen, Denmark; Department of Cardiology, Rigshospitalet, Copenhagen, Denmark; Department of Forensic Medicine, University of Copenhagen, Copenhagen, Denmark; Department of Cardiology, Herlev-Gentofte University Hospital, Copenhagen, Denmark; Department of Cardiology, Herlev-Gentofte University Hospital, Copenhagen, Denmark; Department of Cardiology, Herlev-Gentofte University Hospital, Copenhagen, Denmark; Department of Cardiology, Herlev-Gentofte University Hospital, Copenhagen, Denmark; Department of Cardiology, Herlev-Gentofte University Hospital, Copenhagen, Denmark; The Danish Heart Foundation, Copenhagen, Denmark; Department of Clinical Medicine, Faculty of Health and Medical Sciences, University of Copenhagen, Copenhagen, Denmark; The National Institute of Public Health, University of Southern Denmark, Copenhagen, Denmark; Department of Cardiology, Herlev-Gentofte University Hospital, Copenhagen, Denmark; Department of Clinical Medicine, Faculty of Health and Medical Sciences, University of Copenhagen, Copenhagen, Denmark

**Keywords:** Hypertrophic cardiomyopathy, Atrial fibrillation, Stroke, Oral anticoagulant Therapy

## Abstract

**Aims:**

The treatment of atrial fibrillation (AF) in hypertrophic cardiomyopathy (HCM) can be challenging since AF aggravates symptoms and increases the risk of stroke. Which factors contribute to the development of AF and stroke in HCM remains unknown. The aim of this study was to determine the incidence of AF and stroke in HCM patients and identify the risk factors.

**Methods and results:**

Using Danish national registries, all HCM patients from 2005 to 2018 were included. The association between HCM, incident AF, and stroke was investigated using multivariable Cox proportional hazards analysis. Cumulative incidences were calculated using the Aalen–Johansen estimator. Among the 3367 patients without prevalent AF, 24% reached the endpoint of incident AF with death as a competing risk. Median follow-up time was 4 years. Atrial fibrillation incidence was equal between sexes and increased for patients with ischaemic heart disease [IHD; hazard ratio (HR) 1.33, 95% confidence interval (CI) 1.08–1.63], hypertension (HT) (HR 1.36, 95% CI 1.14–1.67), and obstructive HCM (HR 1.27, 95% CI 1.05–1.52). Seven per cent developed stroke, with no difference detected stratifying for the presence of AF. Sub-analysis revealed that when AF was treated with oral anticoagulants (OACs), stroke was less likely (HR 0.4, 95% CI 0.18–0.86, *P* = 0.02). However, 34% of patients were not receiving adequate anticoagulation following AF diagnosis.

**Conclusion:**

Obstructive HCM, HT, and IHD were associated with increased risk of AF. Prevalent AF alone was not predictive of stroke; however, AF patients treated with OAC were significantly less likely to develop stroke, suggesting that this development is driven by the protective effect of OAC. Despite this, 34% of patients did not receive OAC.

## Graphical Abstract

**Graphical Abstract:**
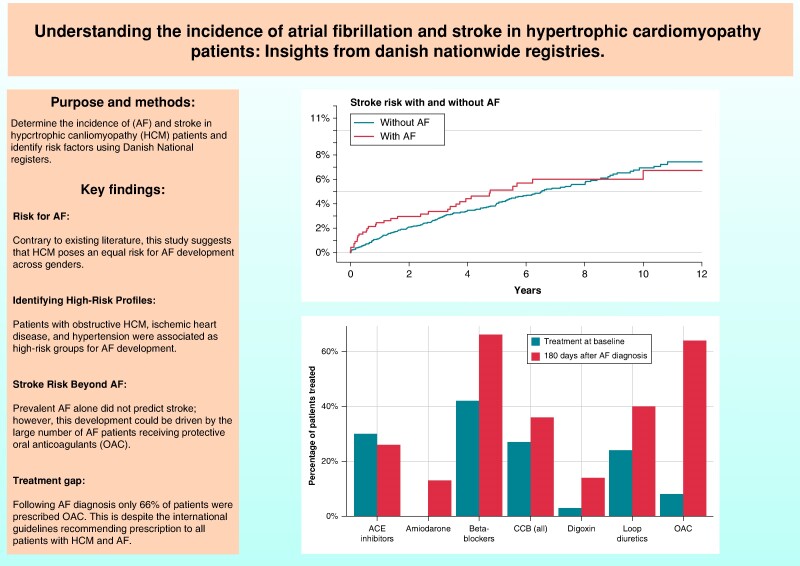

**What’s new?**

- Risk for atrial fibrillation (AF): Contrary to some existing literature, this study suggests that hypertrophic cardiomyopathy (HCM) poses an equal risk for AF development across genders.
- Identifying high-risk profiles: Patients with obstructive HCM, ischaemic heart disease, and hypertension were associated as high-risk groups for AF development.
- Stroke risk beyond AF: While AF is often associated with an increased risk of stroke, this study uncovered a nuanced relationship in HCM patients. Prevalent AF alone did not predict stroke; however, this development could be driven by the large number of AF patients receiving protective OAC treatment.
- Treatment gap: Following AF diagnosis, only 66% of patients were prescribed oral anticoagulants. This is despite the international guidelines recommending prescription to all patients with HCM and AF.

## Introduction

Hypertrophic cardiomyopathy (HCM) affects an estimated 1:500 people worldwide, making it one of the most common genetic cardiovascular disorders.^1–3^

Studies have shown that patients with HCM are at risk of developing a myriad of cardiac complications, in particular atrial fibrillation (AF), stroke, heart failure (HF), and sudden cardiac death.^4–8^ However, many patients with HCM are only mildly symptomatic and may be unaware of their condition, and others only experience symptoms during exertion.^4,6^ This can make diagnostics challenging and complicate further management of the disease, given the severe potential outcome the condition is associated with.

Particularly the management of AF in HCM patients can be a challenge, since AF often aggravates symptoms due to the presence of diastolic dysfunction, necessitating a more aggressive therapeutic approach by clinicians.^5,9,10^ In addition, there is evidence that the presence of AF in the setting of HCM significantly elevates the risk of thromboembolism and stroke when compared to AF patients without HCM.^11–14^ Therefore, most major international guidelines highlight the importance of AF detection in the setting of HCM and recommend the administration of anticoagulant therapy to HCM patients with AF from the first documented onset.^15–17^ However, there is little available data on this topic, and the current practice is based more on scientific consensus than hard scientific evidence.

To improve the level of care for HCM patients, studies examining the development of complications such as AF and stoke as well as identifying potential risk factors for AF and stroke development are warranted. This study was designed as a nationwide cohort study aiming to identify the incidence and risk factors of developing AF and stroke in patients with HCM.

## Methods

### Data sources

All Danish residents are provided with a unique civil registration number, enabling linkage between the individual national Danish registries for research purposes. For this register-based cohort study, the following registries were used: the Civil Registration System, the Danish National Patient Register, and the Danish National Prescription Registry. The Civil Registration System holds data on age, sex, and vital status, and all deaths are registered within 14 days. The Danish National Patient Register holds information on every hospitalization in Denmark since 1978. Primary diagnosis is registered according to the International Classification of Diseases; the 10th revision (ICD-10), since 1994. The database further holds information on operations and procedures performed in Denmark. These procedures have been registered since 1996 and coded according to the Nordic Classification of Surgical Procedures (NCSP) by the Nordic Medico-Statistical Committee.

The Danish National Prescriptions Registry contains data on all prescriptions dispensed by Danish pharmacies since 1994. Pharmaceuticals are registered in accordance with the Anatomical Therapeutic Chemical (ATC) classification system.

### Inclusion

All Danish patients diagnosed with first-time HCM (ICD-10: DI421, DI422) between 1 January 2005 and 31 December 2018 were identified and included in the cohort. The test of this study distinguished between prevalent and incident AF. Prevalent AF includes all patients with an AF diagnosis before inclusion, i.e. date of HCM diagnosis, while incident AF includes all patients diagnosed with *de novo* AF after inclusion. For the analysis of incident AF, only patients without a history of prevalent AF were included, while all patients with newly diagnosed HCM were included into the examination of incident stoke. Patients with incomplete data were excluded.

### Variables

Patient characteristics analysed were sex, age, and type of HCM (obstructive and non-obstructive). For analysis purposes, patients were divided into two groups according to age, patients ages 60 or over and those younger.

Comorbidities analysed included AF, HF, ischaemic heart disease (IHD), ischaemic stroke, chronic kidney disease (CKD), hypertension (HT), and chronic obstructive pulmonary disease and were registered 5 years before study inclusion. ICD-10 codes used for these comorbidities are provided in Supplementary material online, table  *S1*.

Concomitant pharmacotherapy at baseline was defined as any claimed prescription 180 days prior to the date of study inclusion. Medications included in the analysis were beta-blockers, calcium channel blockers (CCBs), angiotensin-converting enzyme (ACE) inhibitors, loop diuretics, spironolactone, oral anticoagulant therapy (OAC), digoxin, and amiodarone. Oral anticoagulant therapy comprised warfarin, phenprocoumon, dabigatran, rivaroxaban, apixaban, and edoxaban. Anatomical Therapeutic Chemical codes used for these pharmaceuticals are provided in Supplementary material online, *Table S2*.

### Statistical analysis

Descriptive tables were employed to describe the study population by morbidity burden with continuous variables reported as medians and interquartile ranges (IQRs) and categorical variables summarized with counts and corresponding percentages. The cumulative incidence of AF and stroke was calculated utilizing the Aalen–Johansen estimator and death accounted for as a competing risk factor. Multivariable Cox proportional hazards analysis was used to examine the association between AF, stroke, and patient characteristics and comorbidities in HCM patients. The analysis was adjusted for sex, age, and comorbidities such as IHD, chronic obstructive pulmonary disease, CKD, and HT.

Statistical analysis and data management were conducted using R statistical software (R Core Team (2021). R: A language and environment for statistical computing. R Foundation for Statistical Computing, Vienna, Austria. URL https://www.R-project.org/.)

### Ethics

According to Danish legislation, retrospective studies using administrative health databases do not require ethical approval in Denmark. The Danish Data Protection Agency has approved the use of registry data, and the current project is registered (approval number: P-2019–408).

## Results

### Demographics

A total of 4030 patients were newly diagnosed with HCM in Denmark between 2005 and 2018. The median age was 66 years (IQR 55–77), and there was a comparable distribution of sexes with females comprising 1867 patients (46%). At the time of initial HCM diagnosis, 1784 (44%) patients had their type of HCM categorized as being obstructive. The prevalence of cardiovascular morbidity was generally high with 49% of patients with prevalent HT, 17% with prevalent AF, 21% with IHD, and 13% with HF. Seven per cent of patients had a previous stroke, and 8% of patients had concurrent chronic obstructive pulmonary disease (*Table 1*).

**Table 1:** Baseline characteristics of study population

	All patients	Patients without AF
Number of patients	4030	3367
Age: median in years [median (IQR)]	66 (54, 77)	65 (51, 75)
Sex: female, *n* (%)	1867 (46)	1597 (47)
HCM type: obstructive, *n* (%)	1784 (44)	1540 (47)
Comorbidities, *n* (%)		
Hypertension	1954 (49)	1496 (44)
IHD	863 (21)	644 (19)
HF	526 (13)	331 (10)
AF	663 (17)	0
Stroke	288 (7)	201 (6)
Chronic obstructive pulmonary disease	313 (8)	231 (7)
CKD	197 (5)	142 (4)
Concomitant pharmacotherapy, *n*, (%)		
Beta-blockers	1425 (35)	1043 (31)
ACE inhibitors	975 (24)	749 (22)
OAC therapy, all	456 (11)	116 (4)
Warfarin	322 (8)	87 (3)
Rivaroxaban	48 (1)	14 (<1)
Apixaban	52 (1)	12 (<1)
Edoxaban	<4 (<1)	<4 (<1)
Dabigatran	41 (1)	<4 (<1)
Phenprocoumon	8 (<1)	<4 (<1)
Loop diuretics	853 (21)	569 (17)
Spironolactone	293 (7)	205 (6)
CCBs, all	1029 (26)	824 (25)
Verapamil	143 (4)	92 (3)
Digoxin	199 (5)	40 (1)
Amiodarone	41 (1)	9 (<1)

For the analysis in incident AF, only patients without previously registered AF were included. This group of 3367 patients was generally comparable to the total HCM cohort in terms of age, distribution between sexes, and comorbidities (*Table 1*).

### Cumulative incidence of atrial fibrillation and stroke

**Figure 1:**
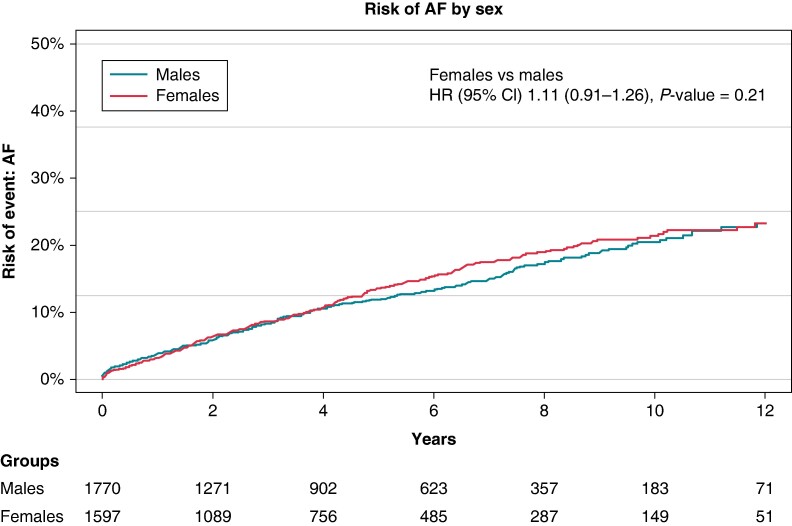
Cumulative incidence of atrial fibrillation—by sex.

Over the observation period of 12 years, 24% of the patients without prevalent AF developed AF with death accounted for as a competing risk. Comparing the cumulative incidence of AF by sex revealed no significant difference between the groups (*Figure 1*).

**Figure 2:**
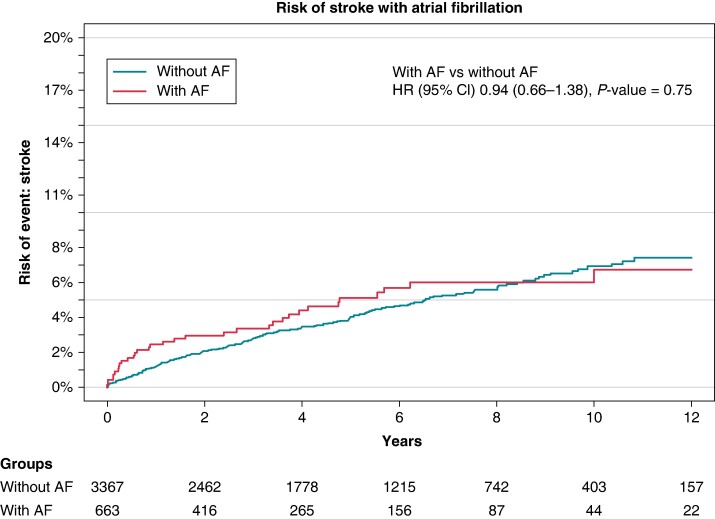
Cumulative incidence of stroke (all patients)—by AF status.

Of all patients included in the study, the cumulative incidence of stroke was 7% over a median follow-up time of 4 years. Patients with prevalent AF were compared to their risk of stroke compared to all patients without AF at time of inclusion. This analysis revealed no significant association for stroke development for either group (*Figure 2*).

**Figure 3:**
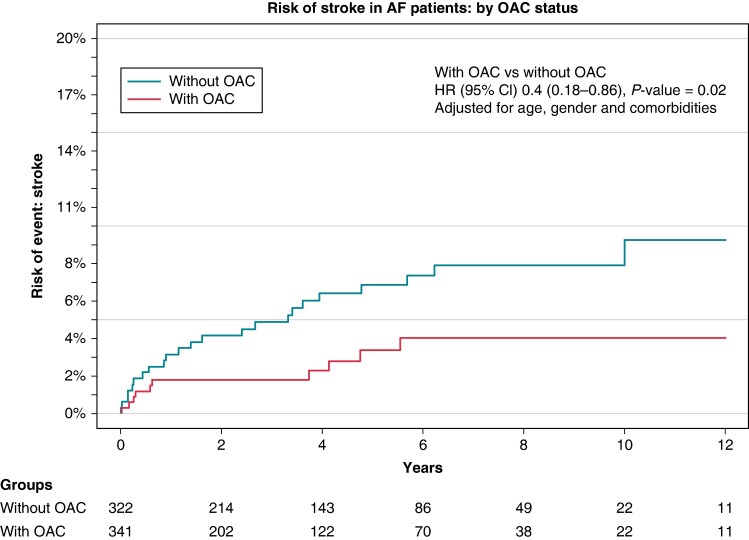
Cumulative incidence of stroke in patients with AF: patients with active (OAC therapy compared to those without.

Of the patients with prevalent AF, 341 (51%) were on active treatment with OACs at time of inclusion. To evaluate whether AF patients on OAC treatment were less likely to develop stroke than AF patients without such treatment, all 663 patients with prevalent AF were divided by status of OAC treatment and compared for their risk of developing stroke or death. This analysis revealed a significantly lower rate of stroke among AF patients in active OAC treatment (4%) compared to those without (10%; HR 0.4, 95% CI 0.18–0.86, *P* = 0.02; (*Figure 3*). Median follow-up time for these patients was 3 years, resulting in an estimated annual risk of stroke of 1.3% for patients on OAC treatment vs. 3.3% for patients without.

### Hazard for incident atrial fibrillation and stroke

**Figure 4:**
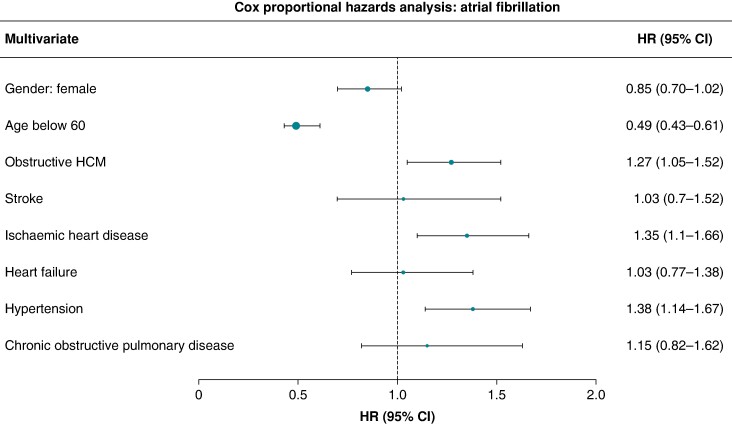
Forrest plot–HR (95% CI) for risk of AF development (patients without prevalent AF)–multivariate analysis.

Further, the risk of AF development was analysed for patient characteristics such as sex and age, as well as prevalence of common cardiovascular comorbidities. The influence of sex on AF development was insignificant, while age below 60 was associated with decreased risk of AF (HR 0.54, 95% CI 0.43–0.67). Similarly, obstructive HCM at inclusion increased the risk of AF in this adjusted analysis (HR 1.27, 95% CI 1.06–1. 53). This was likewise the case for IHD and HT, while presence of HF was not significant (*Figure 4*).

**Figure 5:**
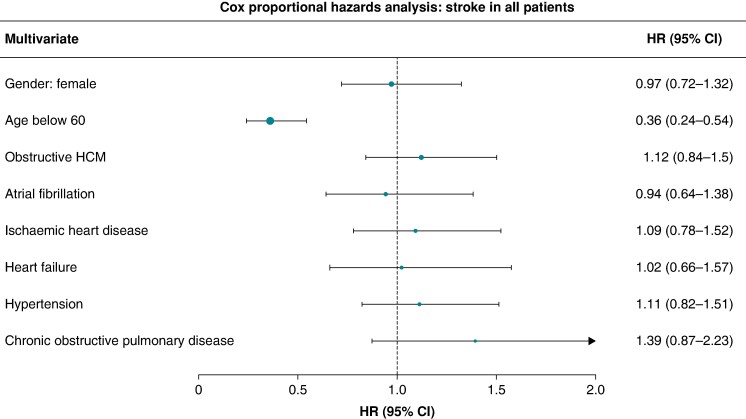
Forrest plot–HR (95% CI) for risk of stroke (all patients)–multivariate analysis.

In the same manner, the risk of stroke development was analysed for all patients. Adjusted for patient characteristics and comorbidities solely, age was shown to significantly modify the risk of stroke, with the HR for patients aged below 60 estimated at 0.36 95% CI 0.24–0.54 (*Figure 5*).

### Changes in therapy following atrial fibrillation diagnosis

**Figure 6:**
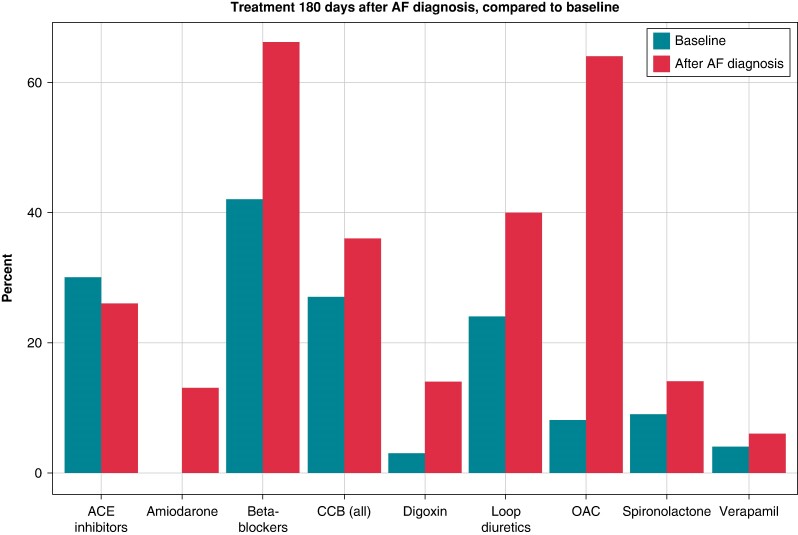
Changes in pharmacotherapy after AF diagnosis.

Finally, to quantify the consequences of AF diagnosis in the context of HCM, changes in pharmacotherapy in 180 days following diagnosis with AF were analysed and compared to these patients’ baseline values. This analysis revealed a large increase in active prescription of antiarrhythmic drugs such as amiodarone (0–13% of patients), digoxin (3–14% of patients), and beta-blockers (42–66% of patients). One hundred and eighty days after diagnosis with AF, 66% of patients were in active OAC treatment (warfarin, 34%; apixaban, 16%; rivaroxaban, 12%; dabigatran, 9%, edoxaban, <1%; phenprocoumon, <1%). Prescription of all types of CCBs increased from 42 to 66%, while usage of verapamil specifically increased from 4 to 6%. Usage of loop diuretics increased 40% of patients, while spironolactone was prescribed to 14% of patients (*Figure 6*). The characteristics of patients who did not receive OAC treatment were compared with those who were treated with OAC revealing no obvious difference between the groups (see Supplementary material online, Table  *S3*).

### Time to atrial fibrillation

The mean time from HCM diagnosis to debut of AF was 1260 days. Time to AF was comparable between the sexes with 1286 days for males and 1231 days for females, while patients aged 60 or older had an earlier debut of AF with 1173 days than patients aged under 60 with 1505 days. Competing prevalent comorbidities, previous stroke, IHD, HF, and even HT had only minimal effect on patient’s time to AF, while chronic obstructive pulmonary disease greatly influenced the time of AF debut with 911 days for those with the disease vs. 1289 days for those without (see Supplementary material online, *Figure S1*).

## Discussion

This nationwide cohort study found that ∼24% of HCM patients without a history of AF developed incident AF during follow-up of median 4 years. Notably, the risk of developing AF was similar between sexes, suggesting that HCM is an equal-opportunity risk factor for AF. However, several key risk factors were identified. Patients with prevalent IHD and HT were at an increased risk of developing AF. In this cohort, AF status alone was not associated with stroke development; however, sub-analysis revealed that AF patients on OAC treatment were significantly less at risk of stroke than AF patients without (HR 0.4). This is notable, since 34% of patients were shown to be without adequate anticoagulation following their diagnosis with AF.

These findings support the conclusion of comparable studies which have estimated AF affecting up to 20% of HCM patients, as well as increasing mortality.^18^

The connection between HT, increased age, and AF development is previously well described and expectedly increased the risk of AF in this HCM population.^19^ However, the connection between IHD and AF, especially in the context of HCM, is less well understood. While an increased risk of AF is well documented in patients following acute myocardial infarction, long-term risk of AF development in patients with IHD is examined to a lesser degree.^20,21^

Sex disparities have been identified both in the incidence of AF and within HCM.^22–26^ While AF has been shown to be more prevalent in men, in this study, the sex distribution was near equal. Sex disparities in AF have in previous studies been linked to a possible influence of hormonal and lifestyle factors such as smoking and alcohol consumption, although the exact mechanism is still a topic of debate.^27–30^ Within HCM, the reason for sex disparities is less clear. While in many studies men are more commonly diagnosed with HCM than women, analysis of this cohort showed a near-equal distribution between sexes. However, women were older and had a higher morbidity burden at initial HCM diagnosis than men. Given that age and comorbidities such as HT are closely linked to AF development, this could serve as a possible explanation for the equal distribution between the sexes in terms of incidental AF.

Furthermore, patients classified as having obstructive HCM at the time of diagnosis also exhibited an elevated risk of AF development. This observation aligns with the clinical understanding that the anatomical and functional characteristics of obstructive HCM, such as left ventricular outflow tract obstruction, can contribute to arrhythmias, including AF. These findings emphasize the need for close surveillance and early intervention in patients with obstructive HCM to mitigate the risk of AF.

Approximately 7% of HCM patients developed a stroke during the study period. Notably, the presence of prevalent AF at the time of inclusion did not significantly predict stroke development, while in the sub-analysis, AF patients in adequate OAC treatment were at a significantly lower risk of developing stroke. This suggests that the equal incidence of stroke among AF patients compared to those without may be driven by the protective effect of OAC. However, also other factors may contribute to the high stroke risk in HCM patients, a finding which is supported by other studies examining stroke risk in the context of HCM.^31,32^ Hypertrophic cardiomyopathy is not only characterized by hypertrophy, but also apical aneurysms, myocardial crypts, and areas of non-compaction, which can potentially be the source of thromboembolism and stroke. Therefore, our results emphasize the importance of comprehensive risk assessment beyond AF in HCM patients to identify those at risk of stroke.

After AF diagnosis, the percentage of patients on OACs increased dramatically; however, roughly a third of patients did not receive OAC treatment, despite the current guidelines recommending prescription of OACs to all HCM patients with AF, regardless of CHADS-VASC score. Given the context of the former analysis which illustrates the impact of OAC treatment in stroke prevention, this finding warrants further exploration.

Certain considerations might help explain this gap. The CHADS-VASC score was only introduced in Europe after its adoption in the 2009 guidelines of the European Heart Rhythm Society and its later inclusion in the 2010 guidelines on the management of AF by the European Society of Cardiology (ESC).^33^ Oral anticoagulant treatment in the context of HCM has been a topic of much debate among researchers and clinicians. The current practice of anticoagulation of all HCM patients, regardless of age and CHAD-VASC, who develop AF was introduced in the ESC guidelines on the management of HCM in 2014.^16^ Since this study includes all patients with a first-time HCM diagnosis from 2006 onwards, a certain gap in OAC treatment can therefore be expected. Additionally, this study does not directly account for relative and absolute contraindications to OAC treatment and other factors which might have guides clinicians in their choice to refrain from prescribing OACs.

Lastly, it is notable to point out that only few patients received amiodarone following AF diagnosis. Further analysis of the cohort revealed that only a fraction of patients (82 patients) would be treated with invasive catheter ablation. It can be argued that the rhythm control strategy in this cohort was overall underutilized.

### Strengths and limitations

Overall, this study provides important information on the risk factors and implications of AF and stroke in patients with HCM. It highlights the importance of early detection and treatment of these complications in order to improve outcomes for patients with HCM.

The strengths of this study include the large sample size, the use of nationwide registry data, and the long follow-up period. However, there are also limitations to consider, including the reliance on registry data, which may be subject to coding errors and incomplete data. Given the relative rarity of HCM, large nationwide databases are well suited for research on this topic. The Danish National Patient Registry and the general completeness of data in the Danish nationwide registries ensure minimal missing data and a complete follow-up.

There is no systematic screening effort in place for AF in the context of HCM. Therefore, the possibility of underdiagnosis remains as well as the possibility of undiagnosed AF prior to diagnosis with HCM which are solely detected due to increased clinical monitoring.

Likewise, absence of AF in stroke patients cannot be excluded in this setting, as in clinical settings, stroke can precede identification of AF. Additionally, the study was unable to account for certain factors that may influence the development of AF, such as lifestyle factors and genetic predisposition. Most notably the utilized registries do not contain data on echocardiography or genetic testing. Hypertrophic cardiomyopathy diagnosis and type classification (obstructive vs. non-obstructive) is therefore solely based on ICD-10 coding within the registries and hereby judgement of the treating physician. In a large validation study examining a variety of cardiovascular diagnoses within the Danish registries, usage of diagnostic codes for HCM was evaluated to have positive predictive value of 90%; however, the sample size for this group was limited.^34^

## Conclusions

Patients with obstructive HCM, HT, and IHD were at increased risk of developing AF. The presence of AF alone was not predictive of stroke development; however, AF patients with sufficient OAC treatment were less likely to develop stroke than those without, highlighting the importance of OAC treatment for AF in the context of HCM. Despite this, 34% of HCM patients remained without adequate anticoagulation following their diagnosis with AF.

## Supplementary Material

euae177_Supplementary_Data

## Data Availability

Data for this study are derived from and accessed through Statistics Denmark. By law, these data are not allowed to be shared. For this reason, data cannot not be made available to other researchers.
