# Supplementary material for: Understanding the incidence of atrial fibrillation and stroke in hypertrophic cardiomyopathy patients: insights from Danish nationwide registries
Source: Europace. 2024 Jun 25;26(7):euae177. doi: 10.1093/europace/euae177 (PMC11242463; doi:10.1093/europace/euae177)

**Supplementary appendix**

**Supplementary table 1:** Diagnostic and procedural codes used to identify comorbidity

| Comorbidity |  |
| --- | --- |
| Atrial fibrillation / Atrial flutter | ICD-10: I48 |
| Chronic Obstructive Pulmonary Disease | ICD-10: J42-J44 |
| Ischemic heart disease | ICD-10: DI20, DI21, DI22, DI23, DI24, DI252, DI255, DI256,  DI258, DI259,  DT822,  DZ951I25 |
| Hypertension | Treatment with more than one anti-hypertensive medication. |
| Congestive heart failure | ICD-10: I50 |
| Ischemic stroke / TIA / systemic embolism | ICD-10: I63, I64, I74, G458, G459 |
| Chronic Kidney Disease | ICD-10: N02-N08, N11-N14, N18, N19, N26, N158-N160, N 162, N163, N164, N168, Q61, E102, E112, E132, E142, I120, M321B |

**Supplementary table 2:** ATC-codes used to define medical therapy

| Oral anticoagulatnt treatment  (Warfarin, phenprocoumon, dabigatran, rivaroxaban, apixaban, edoxaban) | ATC-codes:  B01AA03, B01AA04, B01AE07, B01AF01, B01AF02, B01AF03 |
| --- | --- |
| Beta-blockers | C07A, C07B, C07C, C07D, C07F |
| Calcium channel antagonist | C08C, C08D, C08E, C08G, C09BB, CO9DB |
| Spironolactone | C03D, C03E, C03EB |
| Loop diuretics | C03C, C03EB |
| Non-loop diuretics | C02L, C02DA, C07D, C09XA52, C03A, C03EA, C03B, C03X, C07C, C08G, C09BA, C09DA, C03D, C03E, C03EB |
| ACE inhibitors | C09A, C09B |
| Digoxin | C01AA |
| Amiodarone | C02BD01 |

**Supplementary table 3:** Characteristics and comparison of newly diagnosed AF patients who did / did not receive oral anticoagulation.

|  | Patients without OAC | Patients with OAC | p-value |
| --- | --- | --- | --- |
| Number of patients | 175 | 305 |  |
| Age: median in years (median [IQR]) | 70 [59, 80] | 69 [60, 76] | 0.088 |
| Sex: Female, n, (%) | 82 (47) | 165 (54) | 0.152 |
| HCM type: obstructive, n, (%) | 99 (57) | 168 (55) | 0.825 |
| Comorbidities, n, (%) | | |  |
| Hypertension | 102 (58) | 173 (57) | 0.812 |
| Ischemic heart disease | 53 (30) | 79 (26) | 0.353 |
| Heart failure | 21 (12) | 33 (11) | 0.807 |
| Stroke | 10 (6) | 17 (6) | 1.000 |
| Chronic obstructive pulmonary disease | 18 (10) | 19 (6) | 0.154 |
| Chronic kidney disease | 7 (4) | 9 (3) | 0.725 |

**Supplemetary figure 1:** Time to atrial fibrillation (AF) in days – bar chart.


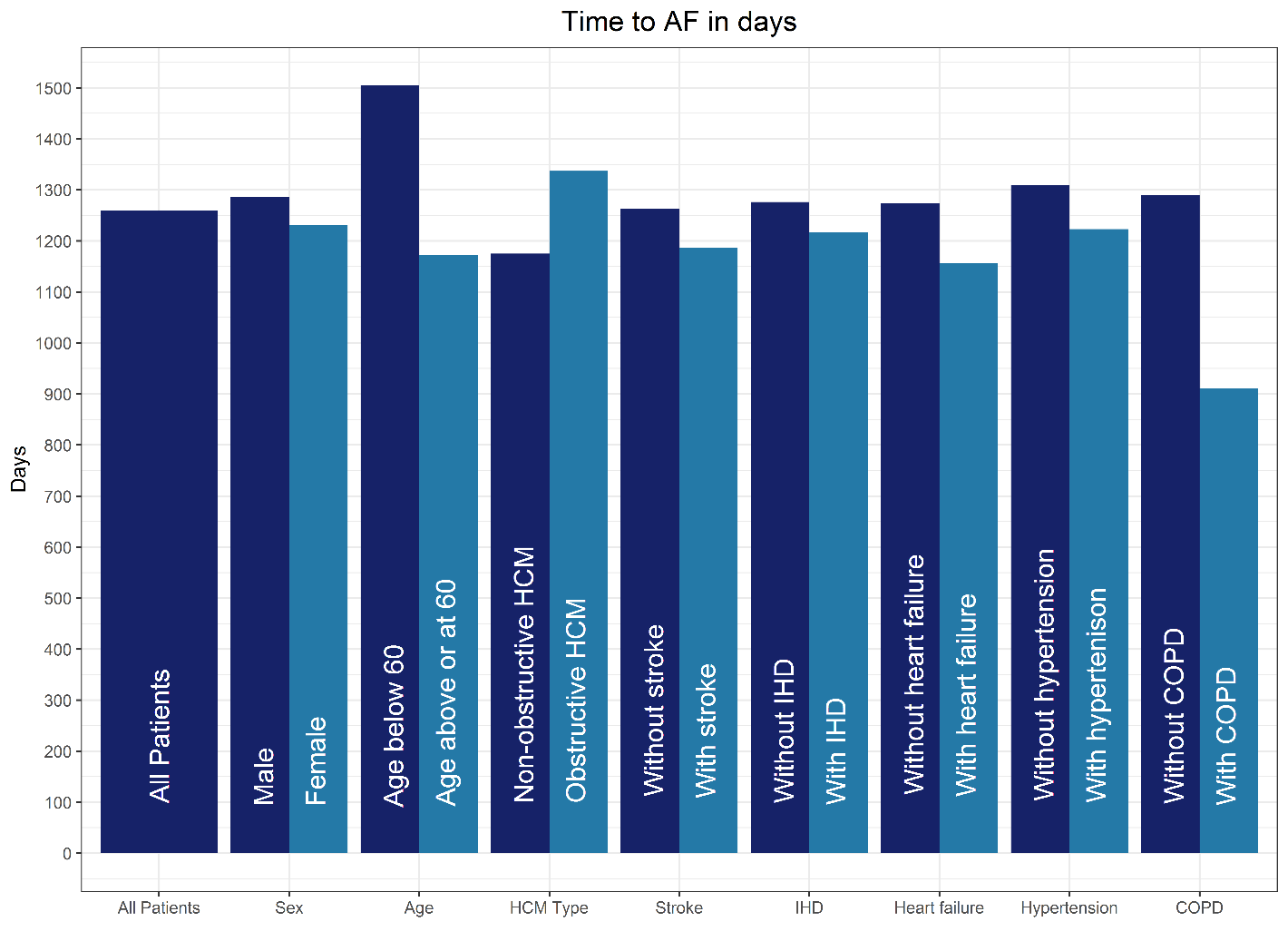

Supplement: euae177_Supplementary_Data [file euae177_supplementary_data.docx]
